# Characterization of Mutants of Human Small Heat Shock Protein HspB1 Carrying Replacements in the N-Terminal Domain and Associated with Hereditary Motor Neuron Diseases

**DOI:** 10.1371/journal.pone.0126248

**Published:** 2015-05-12

**Authors:** Lydia K. Muranova, Stephen D. Weeks, Sergei V. Strelkov, Nikolai B. Gusev

**Affiliations:** 1 Department of Biochemistry, School of Biology, Moscow State University, Moscow 119991, Russian Federation; 2 Laboratory for Biocrystallography, Department of Pharmaceutical and Pharmacological Sciences, KU Leuven, 3000 Leuven, Belgium; UMCG, NETHERLANDS

## Abstract

Physico-chemical properties of the mutations G34R, P39L and E41K in the N-terminal domain of human heat shock protein B1 (HspB1), which have been associated with hereditary motor neuron neuropathy, were analyzed. Heat-induced aggregation of all mutants started at lower temperatures than for the wild type protein. All mutations decreased susceptibility of the N- and C-terminal parts of HspB1 to chymotrypsinolysis. All mutants formed stable homooligomers with a slightly larger apparent molecular weight compared to the wild type protein. All mutations analyzed decreased or completely prevented phosphorylation-induced dissociation of HspB1 oligomers. When mixed with HspB6 and heated, all mutants yielded heterooligomers with apparent molecular weights close to ~400 kDa. Finally, the three HspB1 mutants possessed lower chaperone-like activity towards model substrates (lysozyme, malate dehydrogenase and insulin) compared to the wild type protein, conversely the environmental probe bis-ANS yielded higher fluorescence with the mutants than with the wild type protein. Thus, *in vitro* the analyzed N-terminal mutations increase stability of large HspB1 homooligomers, prevent their phosphorylation-dependent dissociation, modulate their interaction with HspB6 and decrease their chaperoning capacity, preventing normal functioning of HspB1.

## Introduction

Small heat shock proteins (sHsp) form a large family of proteins ubiquitously expressed in practically all kingdoms that play an important role in cellular homeostasis [1–4]. Monomers of sHsp have a comparatively small molecular weight (12–43 kDa) and similar primary structure. They are composed of a conserved α-crystallin domain, a β-sandwich structure, consisting of 90–100 residues that is the hallmark of all proteins belonging to this family [2–5]. The α-crystallin domain is flanked by N- and C-terminal regions that vary in length and amino acid composition and are predicted to have little or no secondary structure. The α-crystallin domain plays an important role in the formation and stabilization of sHsp dimers [6–8] which are the building blocks of larger oligomeric assemblies that are typically formed by these proteins. The C-terminal domain, and in particular the conserved IXI/V motif (detected in the primary structure of certain small heat shock proteins) can interact with the α-crystallin domain of the same or neighboring sHsp monomer possibly modulating stability of dimers and/or the oligomers [9–11]. It is believed that the flexible N-terminal domain exists in multiple environments and provides for formation of large oligomers of certain sHsp often containing more than twenty subunits [10, 12].

The human genome encodes ten members of the sHsp family [13, 14] and certain members of this family (HspB1, HspB5, HspB6 and HspB8) are ubiquitously expressed in practically all human tissues [4, 15]. These proteins participate in a plethora of different functions. For instance, they interact with, and pevent the aggregation of partially denatured proteins which are accumulated after stress, during the course of aging [16] or due to improper protein folding [17]. They can regulate the elimination of denatured proteins by activation of proteolysis in proteasomes or autophagosomes [18]. Small heat shock proteins are also involved in regulation of redox state and protect the cell against oxidative stress [19]. Certain members of the sHsp family possess antiapoptotic activity [20, 21], regulate proliferation [22], stabilize the cytoskeleton [23, 24] and seem to be involved in the regulation of many other vital cellular processes. Since these proteins participate in many different cellular processes, it is not surprising that their mutations have been associated with different congenital diseases such as cataract, desmin-related myopathy, cardiomyopathy, distal hereditary motor neuropathy and Charcot-Marie-Tooth disease [25–27]. In particular, about twenty mutants of HspB1 [25–27] are associated with distal hereditary motor neuropathy and/or type II Charcot-Marie-Tooth disease. The molecular mechanisms underlying development of these diseases in relation to the mutations in HspB1 remain poorly understood. This justifies a detailed investigation of the structure and properties of the different congenital HspB1 mutants. These types of investigations were previously performed on purified HspB1 carrying disease associated point mutations in the α-crystallin domain [28–30] and in the C-terminal region [31]. At the same time mutations of HspB1 in the N-terminal domain that are associated with hereditary motor neuron diseases were not analyzed in detail until now. This paper deals with the investigation of some properties of G34R, P39L and E41K mutants of HspB1 associated with type II Charcot-Marie-Tooth diseases [32] or distal hereditary motor neuropathy [33]. Clinically these mutations result in different manifestations of disease. The G34R and E41K are associated with distal hereditary motor neuropathy (dHMN), whereas mutation P39L is associated with either type II Charcot-Marie-Tooth disease or dHMN. For mutations G34R and P39L the first symptoms of the disease were detected rather late (more than 50 years) as opposed to childhood (less than 10 years) for the E41K mutation and severity of these symptoms were either mild or intermediate [32, 33]. In order to begin to understand the molecular mechanisms underlying these diseases we investigated the physico-chemical properties of these three HspB1 mutants.

## Experimental Procedures

### Cloning and expression of recombinant wild type HspB1 and its N-terminal mutants

cDNA of human wild type HspB1 in pET23b [34] was used for obtaining all N-terminal mutants. Molecular constructs encoding N-terminal mutants of HspB1 were prepared by Eurogen (Moscow) and cloned into pET23b vector at NdeI and XhoI sites. The integrity of the different HspB1 constructs was confirmed by DNA sequencing.

Expression of the wild type HspB1 and its N-terminal mutants was performed by exploiting the leaky regulation of the T7 promoter in the *Escherichia coli* BL21 (DE3) pLysS strain [35]. Briefly, transformed bacteria were grown in 3-fold Luria-Bertani (LB) media at 37°C for 6–8 h followed by growing overnight at 30°C. Bacterial cells were collected by centrifugation and subjected to sonication and ammonium sulfate fractionation (0–40% saturation) as described previously [34]. Further purification was achieved by ion-exchange chromatography using HiTrap Q (HP) column, followed by size exclusion chromatography on Superdex 200 column [34]. Protein preparations were concentrated by ultrafiltration, dialyzed against buffer B (20 mM Tris/acetate, pH 7.6, containing 10 mM NaCl, 0.1 mM phenylmethane sulfonylfluoride (PMSF), 0.1 mM EDTA and 1 mM dithiothreitol (DTT)), aliquoted and stored at -20°C.

The double mutant of HspB6 containing the substitutions C46S/E116C was expressed and purified as described earlier [36].

### Fluorescence spectroscopy

Intrinsic Trp fluorescence of the wild type HspB1 and its N-terminal mutants was measured at 25°C in buffer F (20 mM HEPES/NaOH pH 7.5, containing 100 mM NaCl and 2 mM DTT) at a protein concentration equal to 0.1 mg/ml. Fluorescence spectra were measured using an excitation wavelength of 295 nm (slit width 5 nm) and recorded in the range of 300–400 nm (slit width 5 nm) on Varian Cary Eclipse spectrofluorometer.

Interaction with the fluorescent environment probe bis-ANS was analyzed by titration of the different proteins (0.1 mg/ml) in buffer FF (50 mM phosphate (pH 7.5) containing 100 mM NaCl and 15 mM β-mercaptoethanol (ME)) with increasing amount of a stock solution of bis-ANS (360 μM in buffer FF). Fluorescence was measured using an excitation wavelength of 385 (slit width 2.5 nm) and an emission wavelength of 495 nm (slit width 5 nm). All measurements were performed at 25°C or 37°C. By recording the dependence of fluorescence upon the total concentration of bis-ANS, we determined the apparent binding constant and the number of bis-ANS-binding sites [37].

### Heat-induced aggregation assays

The heat-induced increase of light scattering was measured in buffer F at a protein concentration of 0.3 mg/ml. In this case the protein samples were heated in the range of 20–85°C with a rate of 1°C/min. The cells were illuminated at 340 nm (slit width 2.5 nm) and the signal was recorded at 340 nm (slit width 2.5 nm) on Varian Cary Eclipse spectrofluorometer.

### Limited chymotrypsinolysis

Limited proteolysis was performed in buffer P (20 mM Tris/acetate pH 7.6, containing 10 mM NaCl and 14 mM ME) at 25°C. The wild type HspB1 and its N-terminal mutants (0.5 mg/ml) were incubated with Nα-tosyl-L-lysine chloromethyl ketone-treated chymotrypsin (Sigma) at a weight ratio substrate/chymotrypsin equal to 1000:1 for 0–180 min. The reaction was stopped by the addition of PMSF and the resultant products were analyzed by sodium dodecylsulfate polyacrylamide gel electrophoresis (SDS PAGE) [38]. The rate of proteolysis was determined by plotting the negative logarithm of the ratio (S_t_/S_0_), (where S_t_ and S_0_ are intensity of bands corresponding to uncleaved HspB1 at a given and at zero time respectively), against time.

### Analysis of the quaternary structure

Two methods were used for investigation of the quaternary structures of the different HspB1 constructs. Dynamic light scattering (DLS) experiments were performed with 633 nm radiation using a Zetasizer Nano (Malvern) in DLS buffer (20 mM HEPES/NaOH, pH 7.6, 100 mM NaCl, 2 mM DTT) at 25°C and a protein concentration of 0.3 mg/ml. Each measurement lasted for 15 s and was repeated 10 times and this cycle of measurement was repeated 10 times thus providing accumulation of 100 measurements. The calculations were performed with the Zetasizer software provided by the vendor using the intensity distributions for all subsequent evaluations.

Alternatively the quaternary structure was analyzed by means of size exclusion chromatography.

In this case 150 μl of sample containing variable quantities of protein (10–120 μg) were loaded on Superdex 200 HR 10/30 column equilibrated with buffer S (20 mM Tris/acetate pH 7.6, containing 150 mM NaCl, 0.1 mM EDTA, 0.1 mM PMSF and 15 mM ME). Chromatography was performed at room temperature and at a flow rate of 0.5 ml/min. The column was calibrated with the protein standards thyroglobulin (669 kDa), ferritin (440 kDa), rabbit skeletal muscle pyruvate kinase (240 kDa), rabbit skeletal muscle glyceraldehyde-3-phosphate dehydrogenase (136 kDa), bovine serum albumin (68 kDa), ovalbumin (43 kDa), chymotrypsinogen (25 kDa) and RNAse (13.7 kDa).

### HspB1 phosphorylation

Constitutively active MKK6 protein kinase was incubated with p38 protein kinase for 1 h at 37°C in buffer Ph (25 mM HEPES/NaOH, 25 mM β-glycerophosphate, pH 7.5 containing 2 mM DTT, 20mM MgCl_2_ and 400 μM of ATP). MAPKAP kinase 2 was added to the incubation mixture and incubation at 37°C was continued for another hour. The mixture of activated protein kinases was then divided into four parts and mixed with the wild type HspB1 or the G34R, P39L and E41K mutants each at a final concentration of 0.7 mg/ml. The phosphorylation reactions were incubated at 37°C. At various times a sample was taken and the reaction was stopped either by the addition of EDTA up to the final concentration of 5 mM (for size-exclusion chromatography) or by addition of urea up to the final concentration of 4 M (for urea gel electrophoresis). The extent of HspB1 phosphorylation was determined by quantitative urea-gel electrophoresis [39, 40]. The location of the sites of phosphorylation was determined by means of mass-spectroscopy performed on tryptic peptides of unphosphorylated and completely phosphorylated samples of the wild type HspB1 or its N-terminal mutants. The composition of certain peptides obtained after chymotrypsnolysis was determined by tandem mass-spectroscopy performed on Ultraflex Extreme BRUKER mass spectrometer.

### Formation of heterooligomeric complexes of HspB1 and HspB6

Two different methods were used for analysis of the formation of heterooligomeric complexes between HspB1 and HspB6. In the first case isolated HspB1 (wild type or an N-terminal mutant) and isolated wild type HspB6 were preincubated alone for 30 min at 37°C in the presence of 15 mM DTT in order to reduce their thiol groups. Thereafter the different HspB1 constructs and the wild type HspB6 were mixed in buffer S so that the final concentration of each protein was equal to 0.5 mg/ml. The mixture was divided into two parts and one part was incubated for 1 h on ice where exchange of sHsp subunits is very slow. The second part was incubated at 42°C for 1 h providing for effective subunit exchange. Each sample was loaded on Superdex 200 HR 10/30 column equilibrated with buffer S and operated at a rate of 0.5 ml/min at room temperature. 400 μl fractions were collected and their protein composition was determined by SDS PAGE [38].

Alternatively disulfide crosslinking was used for the analysis of the HspB1–HspB6 interaction. In this case we used the double mutant of HspB6 (C46S/E116C) lacking natural Cys residue in the N-terminal domain and containing a single Cys residue in position homologous to that of Cys137 in HspB1 [36]. Reduced samples of the wild type HspB1 (or its N-terminal mutants) were mixed with reduced Cys-mutant of HspB6 so that the final concentration of each protein was 30 μM per monomer. Isolated proteins or their mixture were incubated for 1 h at 42°C followed by dialysis against 50 mM Tris/HCl pH 7.4, containing 50 mM KCl, 1 mM MgCl2, 0.1 mM PMSF performed at 4, 15 or 37°C. After dialysis the protein samples were mixed with SDS sample buffer lacking ME and subjected to SDS PAGE in the absence of ME.

### Chaperone-like activity

Hen egg white lysozyme, porcine mitochondrial malate dehydrogenase (MDH) or porcine insulin were used as model substrates for the evaluation of the chaperone-like activity. In the case of lysozyme all experiments were performed in buffer L (50 mM potassium phosphate pH 7.4, 0.1 mM EDTA, 50 mM NaCl) at 37°C and at a final concentration of lysozyme equal to 0.14 mg/ml. The lysozyme was freshly prepared by dissolving in 40 mM potassium phosphate pH 4.0 containing 0.1 mM EDTA and subjecting to centrifugation (14.000 g, 10 min). The assay mixture containing buffer L and different quantities of HspB1 or its mutants were preincubated for 10 min at 37°C in the presence of 20 mM DTT in order to ensure reduce thiol groups of this sHsp. The assay was started by the direct addition of lysozyme. The aggregation of reduced lysozyme at 37°C was followed by measuring the optical density at 340 nm using an Ultrospec 3100 Pro spectrophotometer.

All experiments with MDH were performed in buffer M (10 mM sodium phosphate pH 7.4, 2 mM DTT) at 42°C and at the final concentration of MDH equal to 0.2 mg/ml and variable concentrations of different species of HspB1. Heat-induced aggregation of MDH was followed by measuring optical density at 340 nm on Ultrospec 3100 Pro spectrophotometer.

All experiments with insulin were performed by using buffer I (50 mM potassium phosphate pH 8.5, 100 mM NaCl). The incubation mixture contained 170 μl of buffer I, 100 μl of buffer B containing different quantities of HspB1 (or its mutants) and 15 μl of 400 mM DTT. The incubation mixture was preincubated at 37°C for 10 min and reaction was started by addition of insulin dissolved in 2.5% acetic acid up to the final concentration 0.15–0.30 mg/ml. Reduction-induced aggregation of insulin was followed by measuring the optical density at 340 nm on Ultrospec 3100 Pro spectrophotometer.

## Results

### Isolation of the wild type HspB1 and its N-terminal mutants

**Fig 1:**
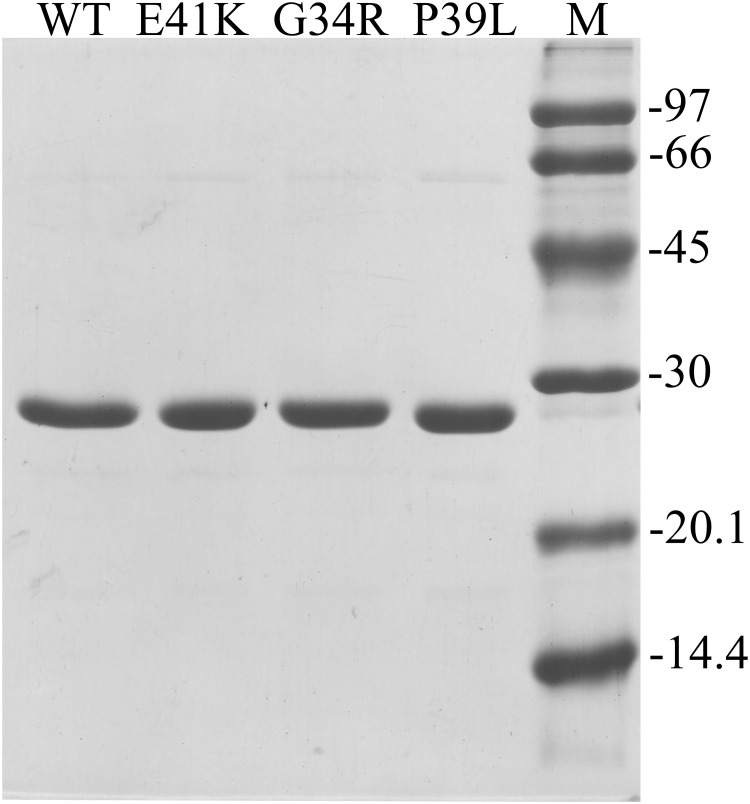
SDS PAGE analysis of the final preparation of the wild type HspB1 and its N-terminal mutants. About 3 μg of each protein was loaded into each track. The positions of the protein markers (track M) and their molecular weight (in kDa) are marked by lines.

The recombinantly expressed WT HspB1 and the three N-terminal mutants could all be readily purified from the soluble fraction of the *E*. *coli* lysate, where none constructs yielded inclusion bodies (data not shown). The purification procedure resulted in homogeneous preparations of the untagged proteins (Fig 1), with a yield of 50–60 mg per 1 l of growth media for each.

### Spectral properties and thermal stability of HspB1 and its mutants

**Fig 2:**
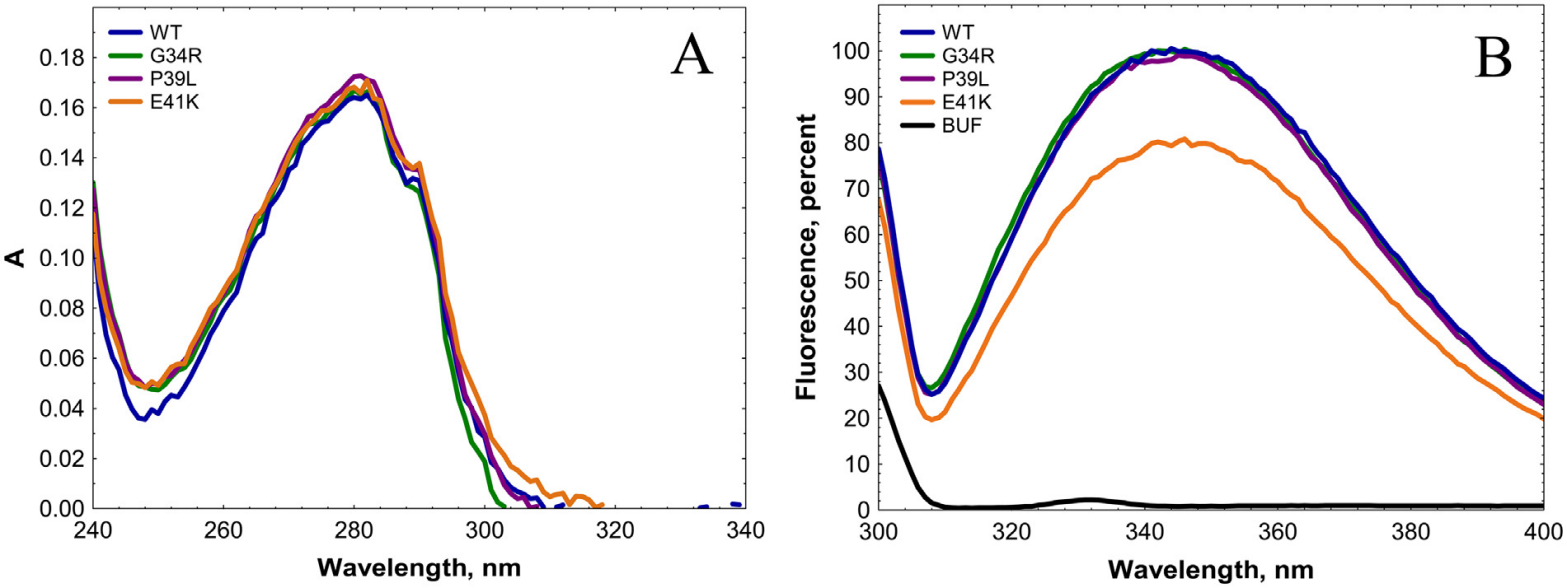
Absorbance (A) and fluorescence (B) spectra of the wild type HspB1 and its N-terminal mutants. Absorption and fluorescence spectra were recorded in buffer F (20 mM HEPES/NaOH pH 7.5, containing 100 mM NaCl and 2 mM DTT) at a protein concentration equal to 0.1 mg/ml. Fluorescence spectra were measured using an excitation wavelength of 295 nm (slit width 5 nm) and recorded in the range of 300–400 nm (slit width 5 nm). The black dotted line in panel B indicates the fluorescence spectrum of buffer.

The absorbance spectra of all analyzed proteins were practically identical (Fig 2A). The fluorescence spectra of all analyzed proteins had a similar shape with a broad maximum located at about 350 nm (Fig 2B). At the same time the amplitude of Trp fluorescence of E41K mutant was about 25–30% lower than that of any other mutants and of the wild type protein (Fig 2B). This is likely due to replacement of the negatively charged glutamic acid by a positively charged lysine in the immediate vicinity of Trp42. Thus, the point mutations analyzed do not induce dramatic changes in the optical properties of HspB1.

**Fig 3:**
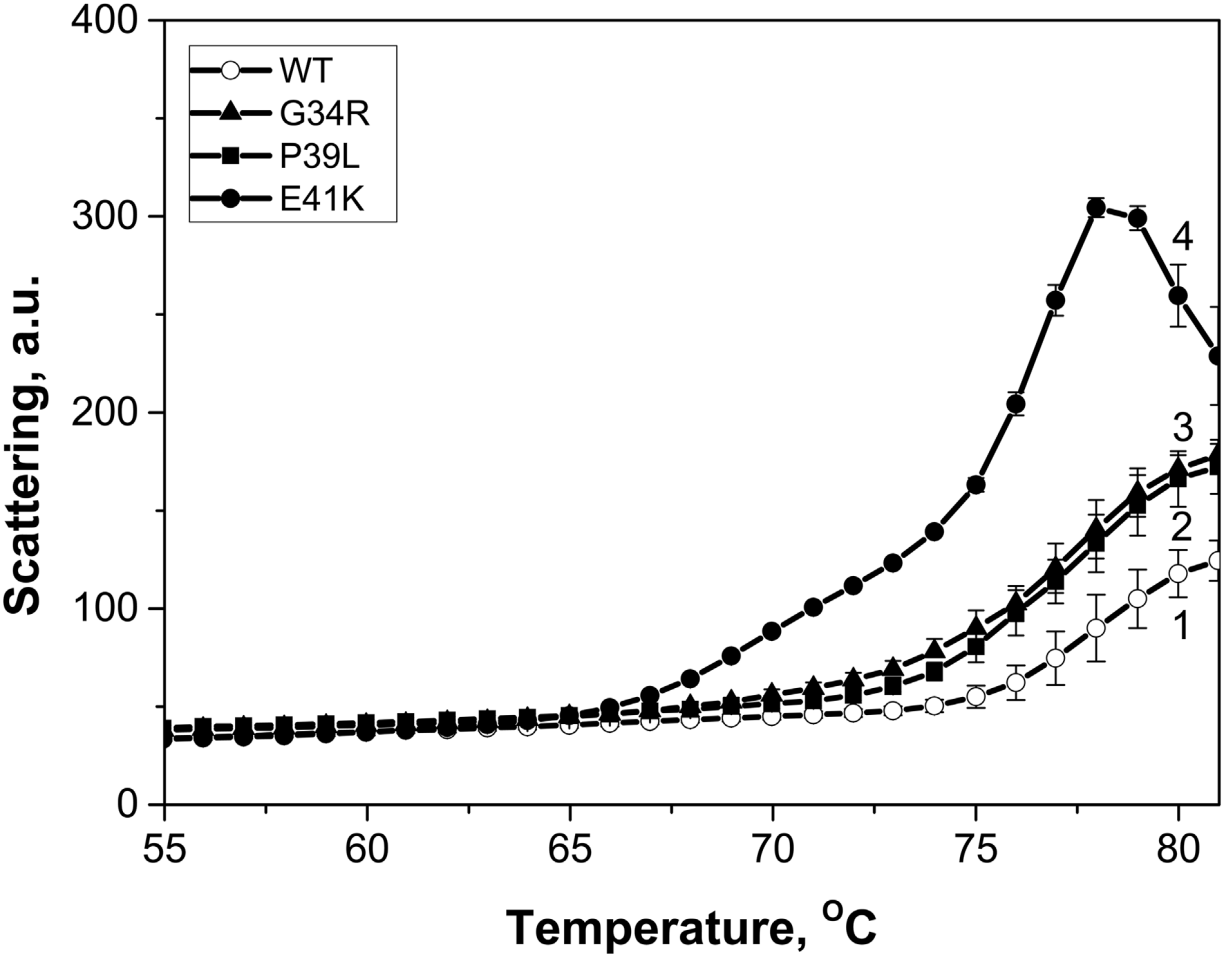
Temperature dependent changes of the light scattering measured at 340 nm of the wild type HspB1 (1) and the G34R (2), P39L (3) and E41K (4) mutants. Protein samples (0.3 mg/ml) in buffer F (20 mM HEPES/NaOH pH 7.5, containing 100 mM NaCl and 2 mM DTT) were heated with a rate of 1°C/min in the range of 20–85°C. The data presented are mean values of four measurements with error bars corresponding to standard deviation.

The temperature-induced aggregation of HspB1 and its mutants was analyzed by recording the light scattering upon heating from 20 to 85°C. As reported earlier [41, 42], heating is accompanied by HspB1 aggregation and increase of light scattering followed by decrease of light scattering which can be due either to the change of density, shape or refractive index of aggregating oligomers. The aggregation started at about 76°C for the wild type HspB1, at about 73°C for the G34R and P39L mutants, and at 62–63°C for E41K (Fig 3). Moreover, the intensity of light scattering of all mutants at elevated temperatures was higher than that of the wild type protein, suggesting a more pronounced aggregation. The data presented indicate that the N-terminal mutants are more sensitive to heating than the wild type HspB1.

### Limited chymotrypsinolysis

**Fig 4:**
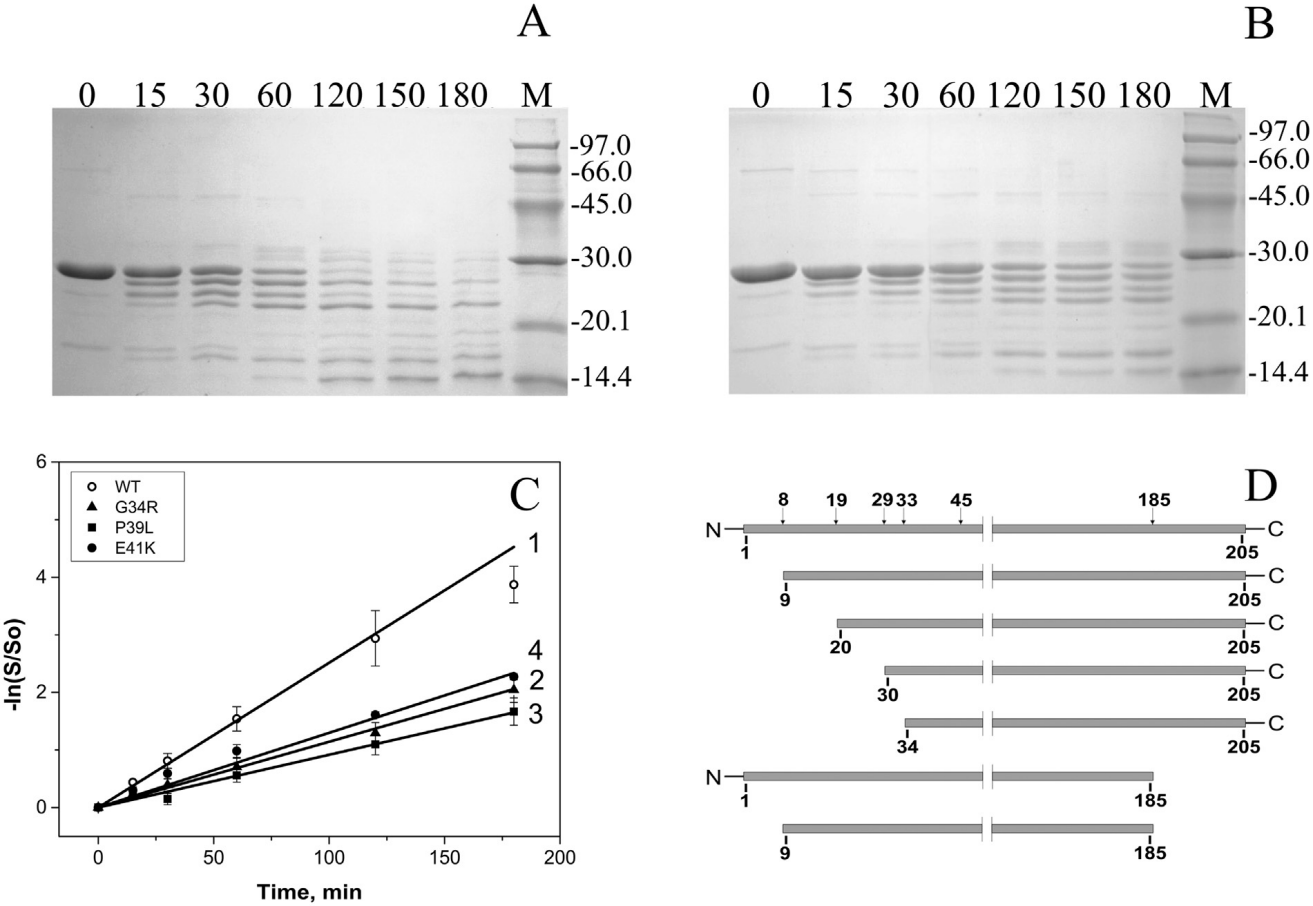
Limited chymotrypsinolysis of HspB1 and its N-terminal mutants. (A) and (B) SDS PAGE analysis of samples obtained after chymotrypsinolysis of the wild type HspB1 (A) and its G34R (B) mutant. Time of proteolysis (min) is indicated above each track. The positions of the protein markers and their molecular weight (in kDa) are indicated by lines. (C) Determination of the apparent rate constant of primary chymotrypsimolysis, as manifested by the intensity decrease of the intact protein band for the wild type HspB1 (1) and its G34R (2), P39L (3) and E41K (4) mutants. The data presented are mean values of five independent measurements with error bars corresponding to standard error of the mean. (D) Location of the most susceptible sites of chymotrypsinolysis in the structure of HspB1 and the cleavage products accumulated at initial stages of chymotrypsinolysis.

Limited proteolysis was used for further analysis of the structure of HspB1 and its N-terminal mutants. Even short incubation of the wild type HspB1 with chymotrypsin (weight ratio HspB1/chymotrypsin equal to 1000/1) was accompanied by accumulation of a series of protein bands with apparent molecular weight of 25, 23 and 21 kDa on SDS PAGE (Fig 4A). Incubation for two hours led to the complete disappearance of the band corresponding to intact uncleaved wild type HspB1 (Fig 4A). At the same time all N-terminal mutants were much more resistant to chymotrypsinolysis and even after prolonged (3 h) incubation remained partially uncleaved (Fig 4B and S1 Fig). The rate at which the band of intact protein diminished was nearly the same for all analyzed mutants and much lower than that for the wild type HspB1 (Fig 4C). Mass spectroscopic analysis revealed that the peptide bonds at Phe8, Phe19, Phe29, Phe33, Trp45 as well as at Leu77 and Phe 185 are most susceptible to chymotrypsinolysis (Fig 4D). As indicated on Fig 4A and 4B initial stages of chymotrypsinolysis are accompanied by truncation of only short peptides of HspB1 thus meaning that aromatic residues located in the very N-terminal or in the very C-terminal part of HspB1 are the primary sites of chymotrypsinolysis. Thus, the data presented show that the point mutations G34R, P39L and E41K inhibit chymotrypsinolysis in the N-terminal part (residues 8, 19, 29 and 33) and/or in the C-terminal part (residue 186) of HspB1. This effect can be probably explained by mutation-induced protection of certain parts of HspB1 monomer inside of large oligomers formed by this protein or, alternatively, an increase in the stability of oligomeric species which are less susceptible to proteolysis.

### Quaternary structure of HspB1 and its N-terminal mutants

**Fig 5:**
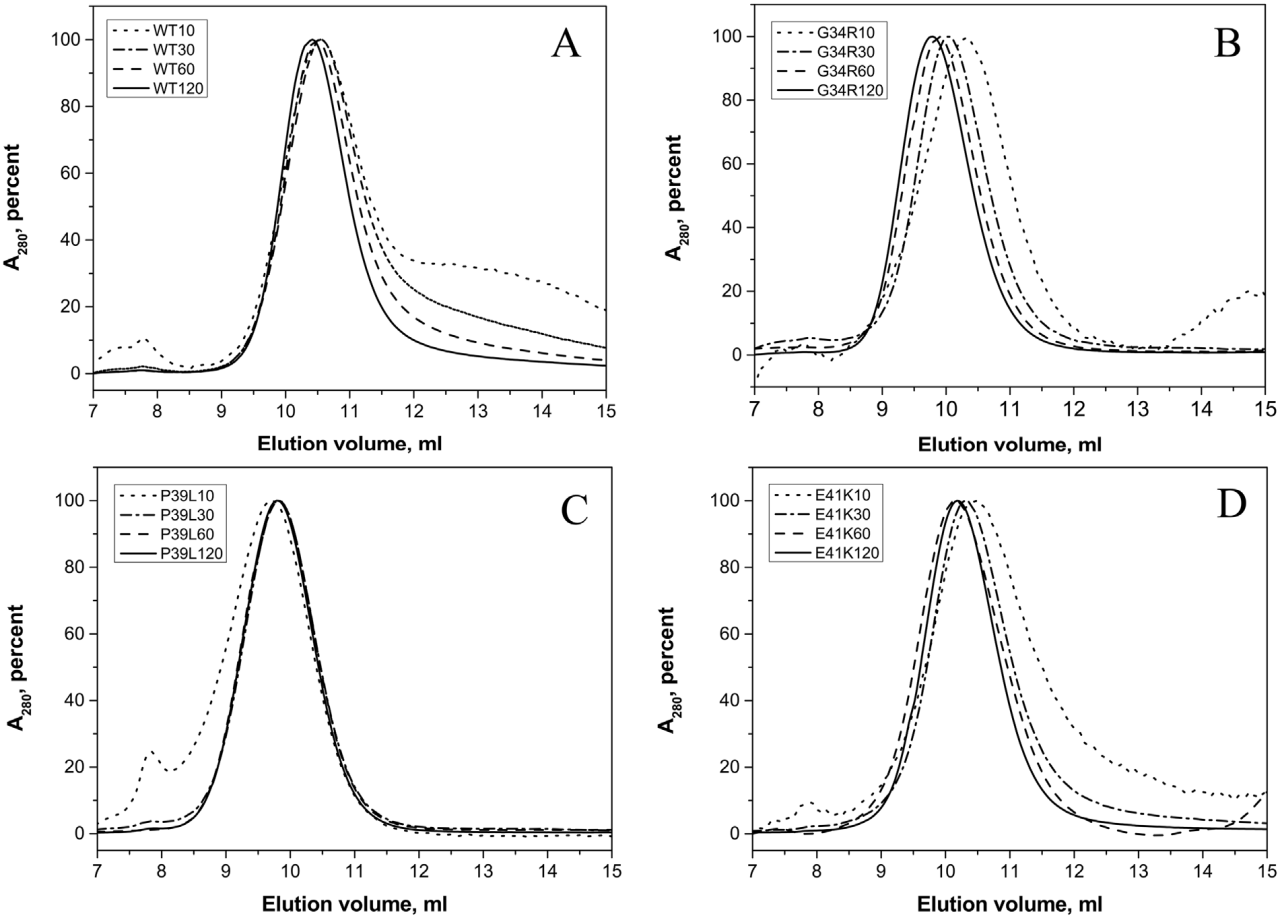
Size-exclusion chromatography of the wild type HspB1 (A) and its G34R (B), P39L (C), and E41K (D) mutants performed in buffer S (20 mM Tris/acetate pH 7.6, containing 150 mM NaCl, 0.1 mM EDTA, 0.1 mM PMSF and 15 mM ME) on a Superdex 200 column at room temperature. 150 μl of sample containing variable quantities of protein (10–120 μg) were loaded on the column and eluted at a flow rate of 0.5 ml/min. Normalized elution profiles obtained after loading on the column 10 (dotted lines), 30 (dash-dotted lines), 60 (dashed lines) and 120 (solid lines) μg of protein are presented.

In size-exclusion chromatography, the wild type HspB1 was eluted as a single peak with an apparent molecular weight of ~530 kDa. The position of the protein peak was practically independent on the quantity of protein loaded on the column (Fig 5A). At low protein loading concentration, positions of the main peaks of G34R and E41K mutants were similar to that of the wild type protein, corresponding to an apparent molecular weight of 530–560 kDa (Fig 5B and 5D). When the quantity of protein loaded on the column was increased the peak of both mutants was shifted towards smaller elution volumes and their apparent molecular weight became equal to~740 kDa for G34R and to ~600 kDa for E41K mutants. The apparent molecular weight of P39L mutant was independent on the quantity of protein loaded on the column and was close to 740 kDa (Fig 5C). These results were further supported by dynamic light scattering on the purified proteins at 0.3 mg/ml. The size of particles formed by the wild type HspB1 was slightly smaller than that of practically all analyzed mutants but the polydispersity of all protein samples analyzed was very similar (Table 1). Thus, the presented data indicate that all mutants form stable oligomers with a size slightly larger than that of the wild type protein or have higher tendency to self-association than the wild type HspB1.

**Table 1:** Size (particle diameter D and polydispersity Pdi) of homooligomers of the wild type HspB1 and its N-terminal mutants as determined by dynamic light scattering.

Proteins	D nm ± s.d. (intensity distribution)	Pdi ± s.d.
Wild type HspB1	16.72 ± 0.94	0.336 ±0.133
G34R	20.97 ± 0.40	0.309 ± 0.035
P39L	21.37 ± 1.14	0.311 ± 0.014
E41K	19.36 ± 0.31	0.309 ± 0.076

### Phosphorylation of HspB1 and its mutants

**Fig 6:**
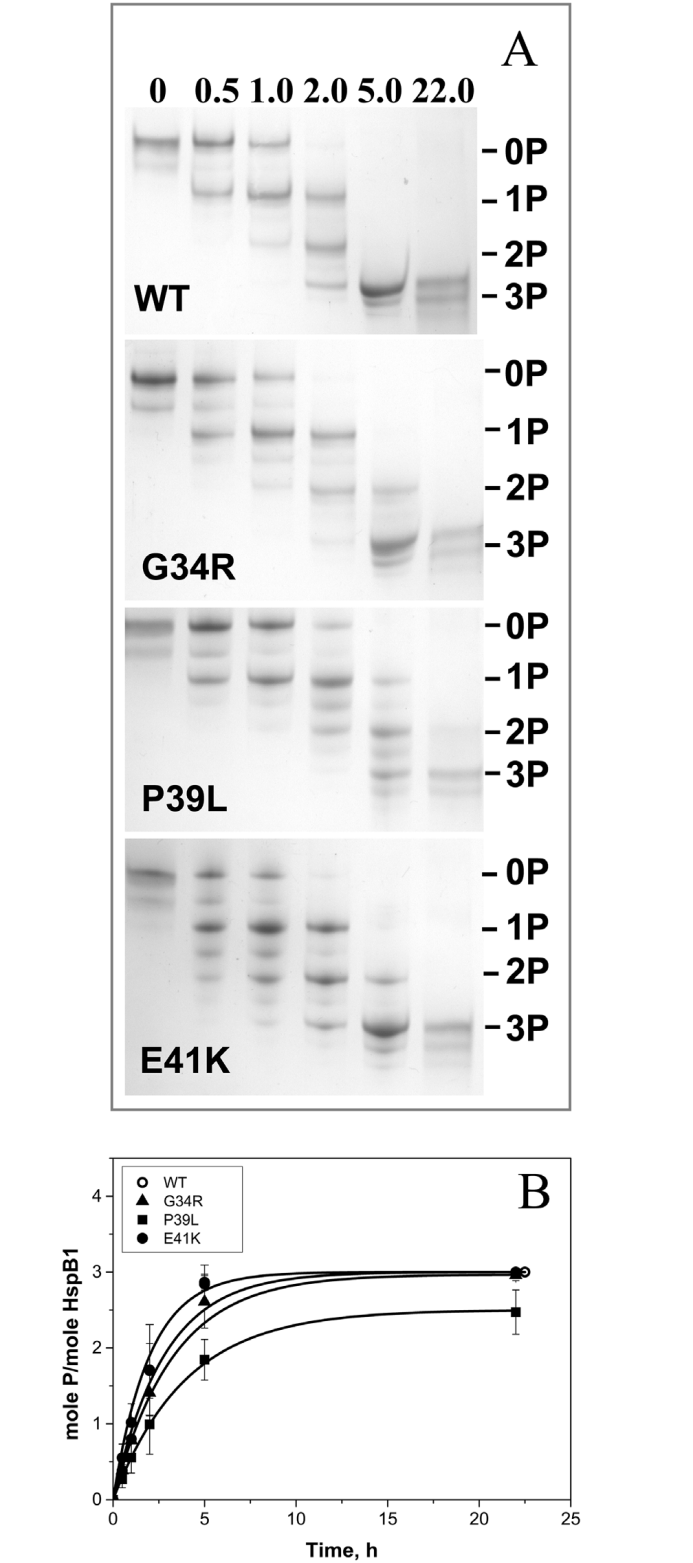
Phosphorylation of the wild type HspB1 and its N-terminal mutants. (A) Urea PAGE of the wild type HspB1 and its mutants after different times of incubation with activated MAPKAP kinase 2. Positions of unphosphorylated, mono-, di- and triphosphorylated species are indicated by lines. The time of incubation is indicated above each track. (B) Kinetics of phosphorylation determined by quantitative densitometry of urea gel electrophoresis. -○-, wild type HspB1, -▲-G34R mutant, -■-P39L mutant, -●-E41K mutant. The data presented are mean values of four independent measurements with error bars corresponding to standard error of the mean.

Human HspB1 can be phosphorylated by MAPKAP kinase 2 at three sites in the N-terminal region: Ser15, Ser78 and Ser82 [43]. Therefore *a priori* the mutations of the N-terminal domain might affect HspB1 phosphorylation. The wild type HspB1 and its N-terminal mutants were phosphorylated by MAPKAP kinase 2 and the extent of phosphorylation was determined by means of quantitative urea-gel electrophoresis (Fig 6). Under the conditions used incorporation of phosphate leads to an increase in electrophoretic mobility and therefore the triple phosphorylated HspB1 sample possesses the highest mobility, whereas species containing one or two mole of phosphate per mole of protein possess intermediate mobility [40]. Sometimes (especially in the case of P39L and E41K mutants) we observed additional bands with electrophoretic mobilities intermediate between those of mono-, di- and triphosphosphorylated samples of HspB1 (Fig 6). These bands seem to originate from incomplete dissociation of oligomers of mutated HspB1 (discussed below). Prolonged incubation of HspB1 with MAPKAP kinase 2 resulted in incorporation of three moles of phosphate per mole (Fig 6B) and the rate of phosphorylation of G34R and E41K was similar to the rate of phosphorylation of the wild type protein, whereas the rate and extent of phosphorylation of P39L was slightly lower (Fig 6B).

**Fig 7:**
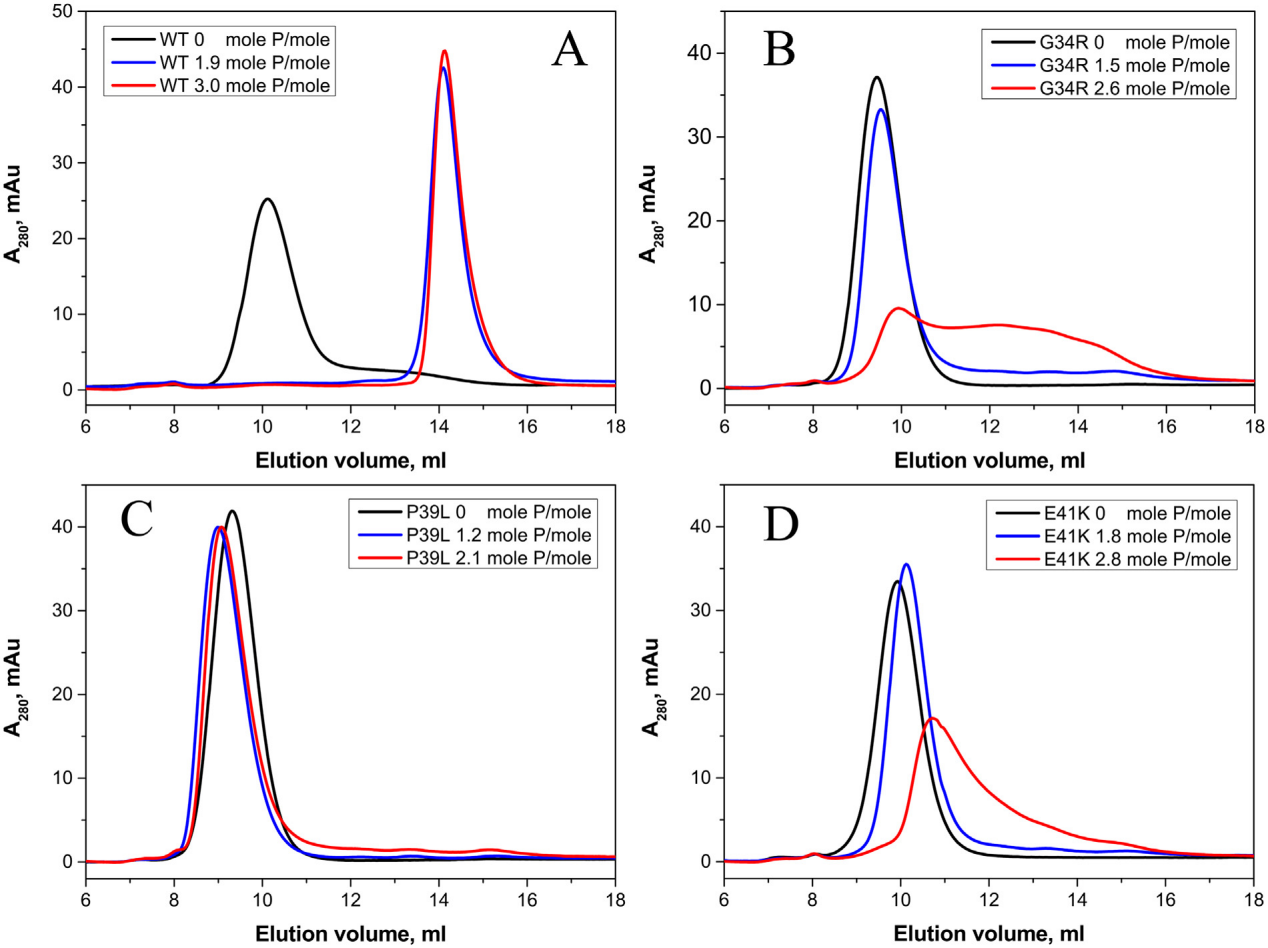
Size-exclusion chromatography of the wild type HspB1 and its G34R, P39L and E41K mutants before phosphorylation (black curves) and with different levels of phosphorylation (blue and red curves) run at a flow rate of 0.5 ml/min in buffer S (20 mM Tris/acetate pH 7.6, containing 150 mM NaCl, 0.1 mM EDTA, 0.1 mM PMSF and 15 mM ME) on a Superdex 200 column at room temperature. The extent of phosphorylation, which was determined by quantitative densitometry of urea PAGE gels, is indicated on each panel.

Phosphorylation catalyzed by MAPKAP kinase 2 induces the dissociation of large oligomers and accumulation of small oligomers (dimers or tetramers) of the wild type HspB1 [44]. Partial dissociation of large oligomers of the wild type HspB1 is observed even after incorporation of less than 1 mole phosphate per mole of HspB1 [30, 44] (S2 Fig). We analyzed the effect of phosphorylation on the oligomeric structure of the wild type HspB1 and its N-terminal mutants. As expected, phosphorylation up to 1.2–1.6 mole per mole of the wild type protein was accompanied by complete dissociation of large oligomers and accumulation of only small oligomers of the wild type HspB1 (Fig 7). At equivalent phosphorylation levels the oligomeric state of the N-terminal mutants was not changed and they remained in the form or large oligomers (Fig 7). At phosphorylation levels exceeding 2 moles of phosphate per mole of protein we detected only partial dissociation of large oligomers of G34R and E41K mutants, whereas large oligomers of the P39L mutant remained practically unchanged (Fig 7 panels B, D and C respectively). There is a possibility that the mutations affect the specificity of MAPKAP kinase 2 or the other kinases present in the activating mixture and that other sites on HspB1 were phosphorylated leading to the observed results. However, this was disproved as mass-spectroscopy analysis of tryptic peptides showed that the same sites were phosphorylated in the wild type HspB1 and its P39L mutant (S3 Fig and S1 Table). Thus, the data presented indicate that the mutations in the N-terminal domain partially or completely prevent phosphorylation-induced dissociation of large oligomers of HspB1.

### Formation of heterooligomeric complexes of HspB1 with HspB6

**Fig 8:**
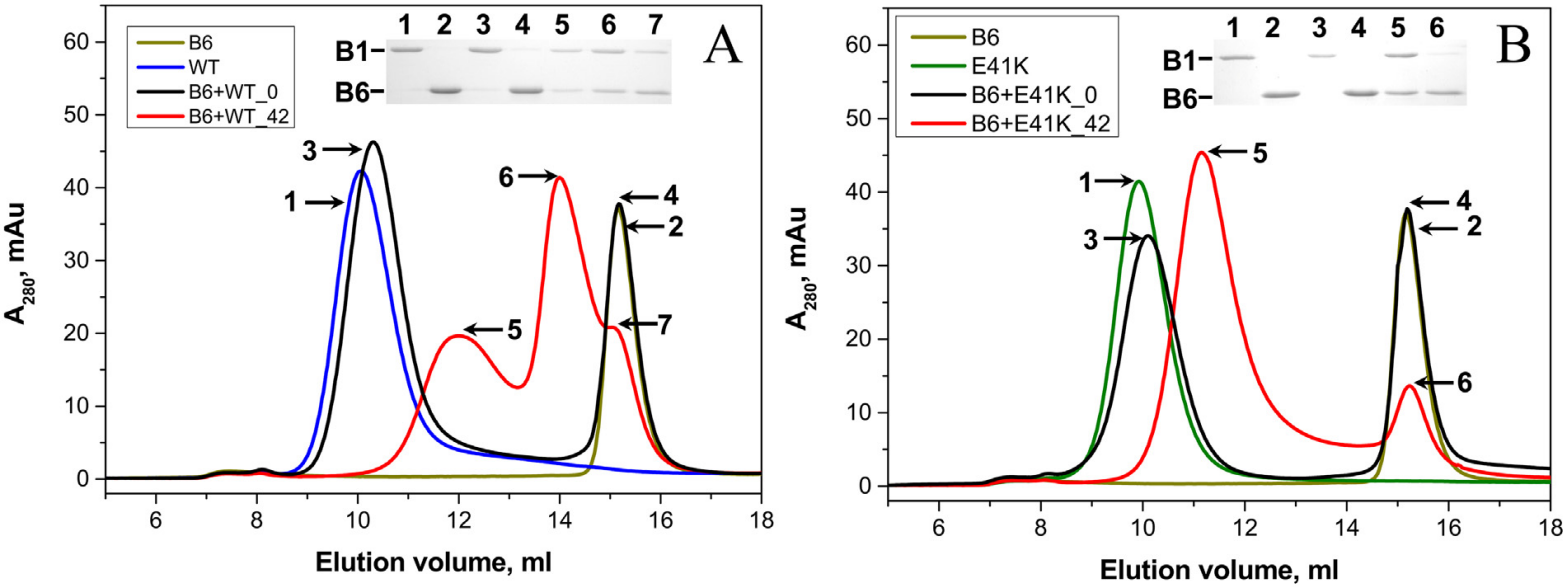
Interaction of the wild type HspB1 (A) and its E41K mutant (B) with the wild type HspB6 analyzed by means of size-exclusion chromatography performed at room temperature on a Superdex 200 column. On each panel elution profiles of the isolated wild type HspB1 (blue line), isolated E41K mutant (green line) and isolated HspB6 (dark yellow line) are presented. The elution profiles of the mixture of the two proteins preincubated at 4 and 42°C are shown as black and red lines respectively. The protein composition of samples collected at positions marked by the numbered arrows is presented in the insert. Positions of HspB1 (B1) and HspB6 (B6) are marked by lines.

The wild type HspB1 effectively interacts with HspB6 forming two types of heterooligomeric complexes with apparent molecular weights of ~100 and ~300 kDa [29, 30, 45](Fig 8A and S4 Fig). The N-terminal mutants were also able to interact with HspB6. As in the case with the wild type HspB1, this interaction was dependent on the temperature of incubation, and we observed formation of heterooligomeric complexes only after preincubation of the mixture of HspB1 mutants and HspB6 at elevated temperature (Fig 8B). Under the analyzed conditions the N-terminal mutants formed only one type of heterooligomeric complex with HspB6 with apparent molecular weight between 370–380 kDa for E41K and G34R mutants and 420 kDa for P39L mutant (Fig 8B, S4 Fig).

**Fig 9:**
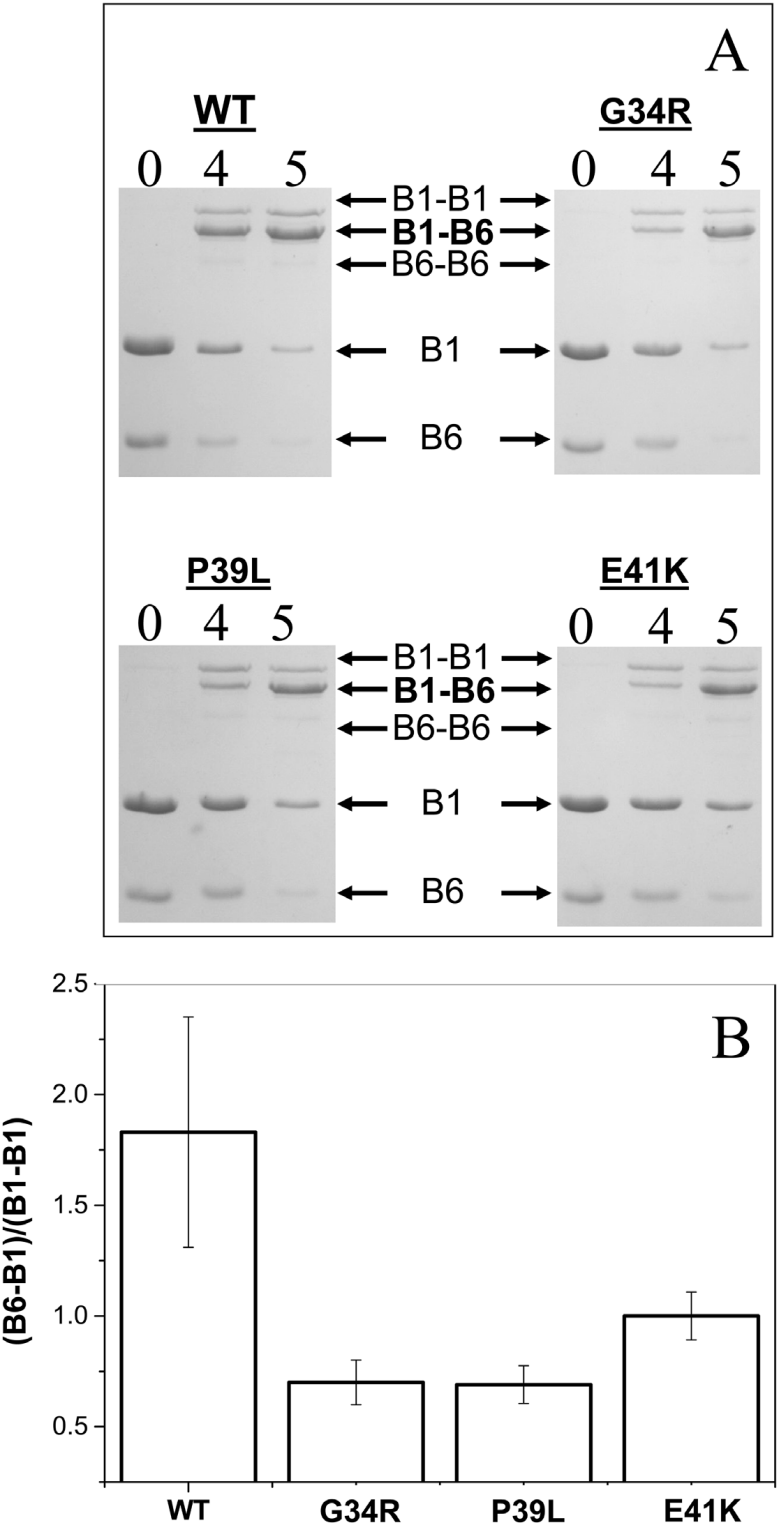
Disulfide crosslinking of HspB1 and the Cys mutant of HspB6 inside heterooligomeric complexes. (A) SDS PAGE analysis, performed under non-reducing conditions, of heterooligomeric complexes formed between the Cys-mutant of HspB6 and the wild type HspB1 and its G34R, P39L and E41K mutants. The positions of monomeric HspB6 (B6) and HspB1 (B1), disulfide crosslinked homodimers of HspB1 (B1–B1) and crosslinked heterodimers of HspB1 and HspB6 (B1–B6) and of molecular weight standards are indicated by arrows. Time of dialysis (h) is indicated above each track. (B) Ratio of the peak corresponding to crosslinked HspB1-HspB6 (B1–B6) to that of crosslinked HspB1 (B1–B1) after dialysis for 4 h. The data presented are mean values of four independent measurements with error bars corresponding to the standard error of the mean.

Disulfide crosslinking was also used for investigation of the interaction of the wild type HspB1 and its N-terminal mutants with HspB6. In this case we mixed HspB1 (and its mutants) with the so-called Cys mutant of HspB6, containing a single Cys residue in a position homologous to that of Cys137 of HspB1 [36]. Mild oxidation leads disulfide crosslinking of Hsp monomers within the homo- or heterooligomeric complexes. In preliminary experiments we failed to detect any changes in kinetics of disulfide crosslinking of monomers inside of the wild type HspB1 homooligomers or any of its N—terminal mutants (S5 Fig). At the same time disulfide bridged heterodimers formed between HspB6 and any of the HspB1 N-terminal mutants were accumulated more slowly than the corresponding heterodimers between HspB6 and the wild type HspB1 (Fig 9). Qualitatively similar results were obtained if disulfide crosslinking was performed at 4, 15 (data not presented) or 37°C. Thus the mutations in the N-terminal domain affect either kinetics of subunits exchange or the probability of crosslinking HspB1 and HpsB6 in heterooligomeric complexes.

### Chaperone-like activity

**Fig 10:**
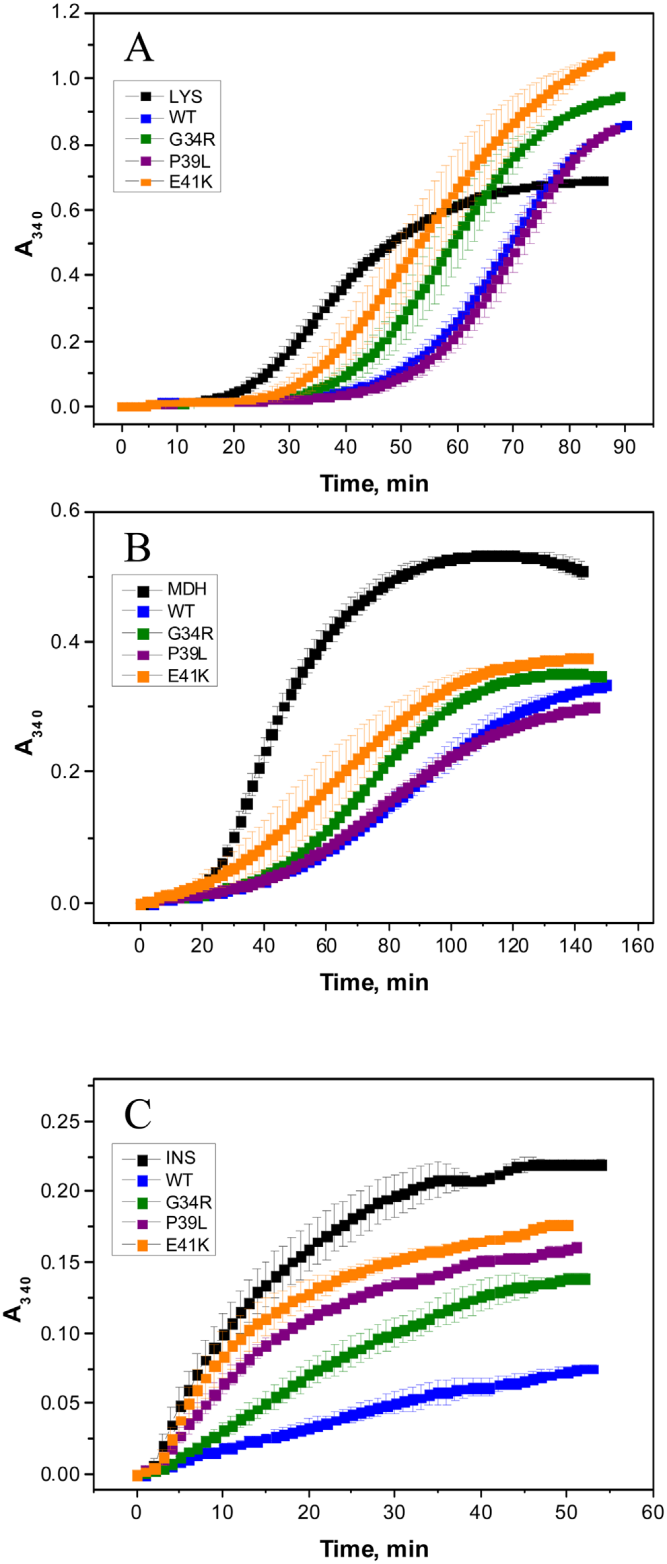
Chaperone-like activity of the wild type HspB1 and its N-terminal mutants with lysozyme (LYS)(A), malate dehydrogenase (MDH) (B) and insulin (INS)(C) as model protein substrates. Aggregation of denatured protein substrates was followed by recording the optical density at 340 nm. The curves labeled LYS, MDH and INS correspond to aggregation of isolated protein substrates in the absence of HspB1, whereas the curves labeled WT or by a mutant name correspond to aggregation in the presence of the different HspB1 constructs. The concentration of lysozyme was equal to 0.14 mg/ml and the concentration of HspB1 and its mutants in this experiment was equal to 0.20 mg/ml respectively. The concentration of MDH was equal to 0.20 mg/ml and that of the HspB1 variants was equal to 0.02 mg/ml. The concentration of insulin was equal to 0.15 mg/ml and that of the HspB1 variants was equal to 0.03 mg/ml. Data are representative of at least three independent experiments with error bars corresponding to the standard error of the mean.

Three different model substrates, namely lysozyme, malate dehydrogenase (MDH) and insulin were used for estimation of the chaperone-like activity of HspB1 and its N-terminal mutants. Reduction of disulfide bonds is accompanied by denaturation and aggregation of lysozyme (Fig 10A). The wild type HspB1 delayed the appearance of light scattering aggregates of reduced lysozyme by 20 minutes (Fig 10A). The chaperone-like activity of the P39L mutant was comparable with that of the wild type protein, whereas the chaperone-like activity of G34R and especially the E41K mutant was lower than that of the wild type HspB1.

Heating induces denaturation and aggregation of MDH and the wild type HspB1 retarded aggregation of this model protein substrate (Fig 10B). Based on final absorbance the G34R and E41K mutants were moderately less effective in retardation of MDH aggregation, whereas the chaperone-like activity of P39L mutant with this model protein substrate was comparable to that of the wild type protein.

Finally, in the case of reduction-induced aggregation of insulin we found that all N-terminal mutants possessed lower chaperone-like activity than the wild type HspB1. With this model protein substrate E41K mutant had the lowest chaperone-like activity followed by the P39L and G34R mutants (Fig 10C).

The data presented indicate that as a rule the chaperone-like activity of the N-terminal mutants is less than that of the wild type protein, however, the chaperone-like activity of N-terminal mutants is dependent on the nature of model protein substrate and can be comparable with that of the wild type protein with one model substrate and at the same time markedly smaller in the case of another model protein substrate.

**Fig 11:**
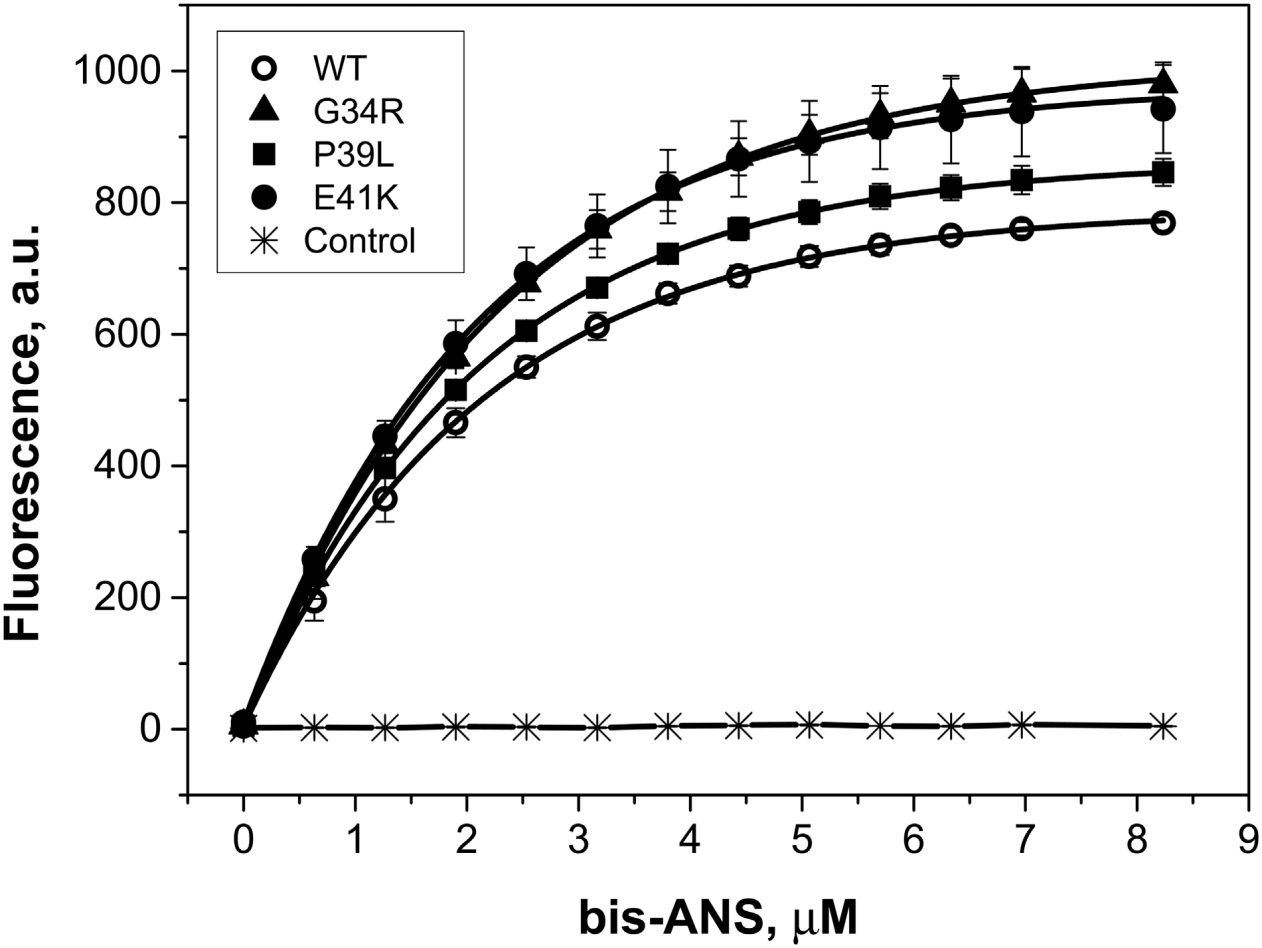
Titration of the wild type (WT) HspB1 and its N-terminal mutants with the environmental probe bis-ANS. Protein samples (0.1 mg/ml) in buffer FF (50 mM phosphate (pH 7.5) containing 100 mM NaCl and 15 mM β-mercapthoethanol (ME)) were titrated with a stock solution of bis-ANS (360 μM in buffer FF). Fluorescence was measured using excitation wavelength of 385 (slit width 2.5 nm) and an emission wavelength of 495 nm (slit width 5 nm). All measurements were performed at 25°C. The titration curve of the buffer in the absence of any protein is marked as control. The data presented are mean values of six independent measurements with error bars corresponding to standard deviation.

It is assumed that the chaperone-like activity is at least partially dependent on hydrophobic interactions between small heat shock proteins and their protein substrates [2, 46, 47]. Therefore it seems reasonable to compare hydrophobic properties of the wild type HspB1 and the different N-terminal mutants. Titration with the environmental sensitive hydrophobic probe bis-ANS is accompanied by a significant increase in fluorescence (Fig 11). Non-linear fitting of the titration curves revealed no significant differences in the apparent binding constant or the number of bis-ANS-binding sites, however the maximal intensity of fluorescence of the complexes formed by bis-ANS and HspB1 mutants was larger than that of the complexes formed by bis-ANS and the wild type HspB1 (Fig 11). This effect can be due to the change in the environment of bis-ANS-binding sites in the wild type HspB1 and its N-terminal mutants. These changes can be induced by replacement of neutral (G34) or negatively charged residues (E41) by positively charged arginine or lysine in G34R and E41K mutants, respectively. At the same time the data presented indicate that decreased chaperone-like activity of the N-terminal mutants is not due to decrease in their hydrophobicity.

## Discussion

The N-terminal domain of vertebrate small heat shock proteins is generally considered as being poorly conserved at the sequence level and predicted to contain little or no secondary structure [12]. This is reflected in available structures, in particular those from vertebrates for which no full-length crystal structure exists [12]. At present, within this subphylum the 3D structure is described only for the isolated α-crystallin domain with a few additional amino acid residues on its N- and C-terminal ends [6–8, 48], while no full-length vertebrate sHsps could be crystallized thus far. Despite the apparent lack of structure the N-terminal domain plays an important role both in stabilization of oligomeric structure and in defining the chaperone-like activity of human sHsps [12, 49, 50]. This dual role can complicate detailed analysis of different parts of the N-terminal domain in sHsp functioning. This complication was recently overcome by analysis of functional role of different parts of the N-terminal domain of human HspB6 predominantly presented in the form of stable dimers [51].

**Fig 12:**
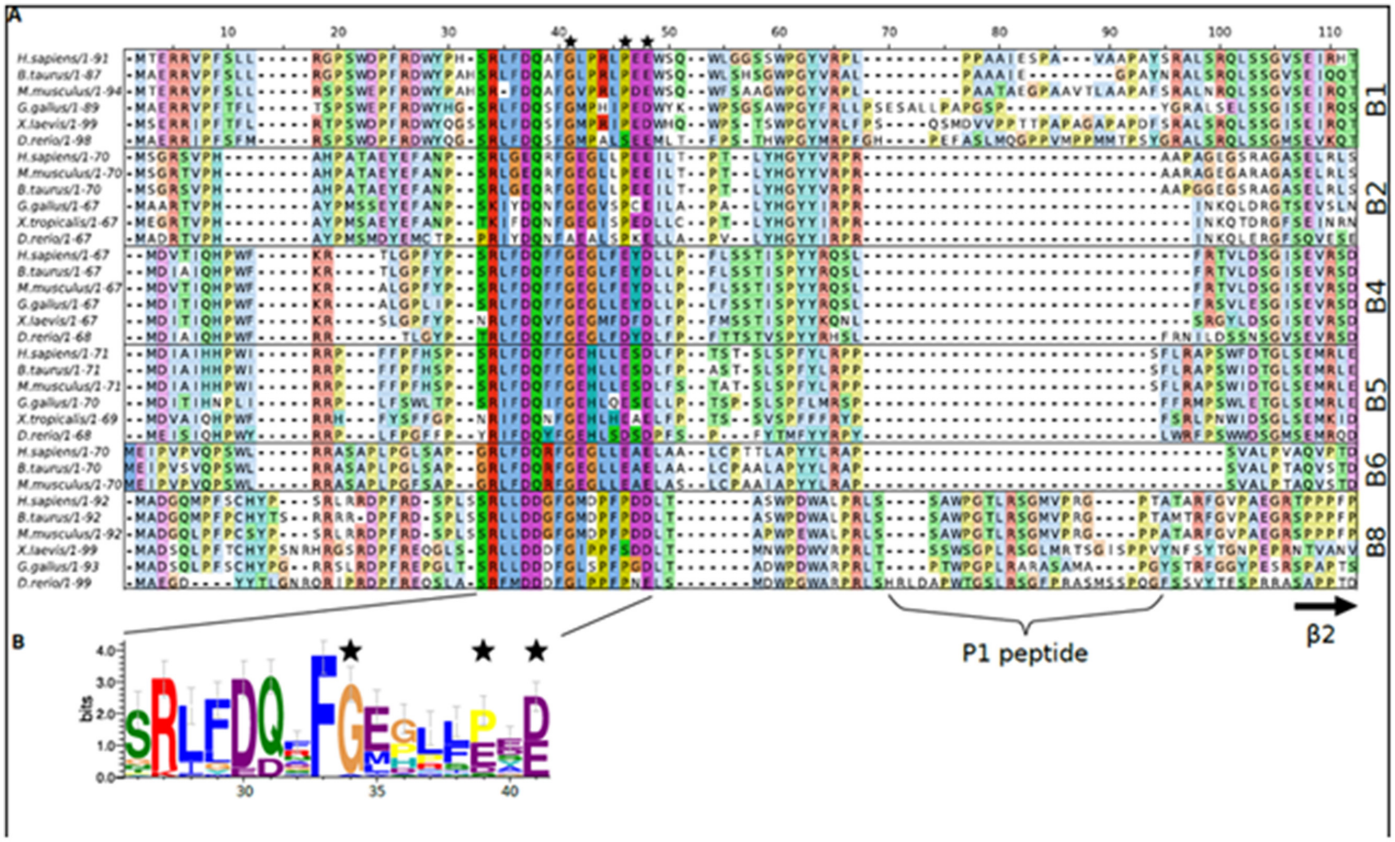
Sequence analysis of the N-terminal domain of different vertebrate sHsp homologues. (A) Multiple sequence alignment of the N-terminal domain of sHsp from selected vertebrate species. Identified sequences corresponding to the N-terminal domain of human HspB1, HspB2, HspB4, HspB5, HspB6 and HspB8 were aligned against their orthologues found in bovine, rodent, Xenopus and zebrafish with Muscle [54]. Using Jalview [55] the aligned sequences were grouped based on their UniProt annotation and each group was colored using the ClustalX scheme. The commonly conserved sequence motif, corresponding to residues 26–41 in HspB1, is darkly colored across the alignment. The P1 peptide insert and the residues corresponding to β2 of the α-crystallin domain, as determined from available crystal structures, are highlighted below the alignment. (B) Sequence logo representation of the conserved sequence motif. Residues are colored according to the ClustalX scheme. Numbering of the residues is according to the HspB1 sequence. The image was initially created using weblogo 3 [56]. In both panels the position of the three mutated residues in HspB1 characterized here are highlighted by a black star.

Provisionally four different regions can be described in the N-terminal domain of HspB1 (Fig 12A). The first encompasses approximately 25 residues of the immediate N-terminus of this sHsp, HspB6 and HspB8 and about 20 N-terminal residues in HspB2 and the two α-crystallins (HspB4 and HspB5). This region is enriched in hydrophobic/aromatic and positively charged residues and seems to be responsible for the interaction of sHsp with different protein ligands. Mutations of certain residues or partial deletion of this region [51] were accompanied by a decrease of chaperone-like activity. Deletion of the first 15 residues did not affect the oligomeric structure of Chinese hamster Hsp27 (ortholog of human HspB1), whereas a deletion of the first 23–30 N-terminal residues was accompanied by dissociation of large oligomers of this protein [52]. Mutations in this region also affected the oligomeric structure of HspB5 [53].

The second region restricted to residues 26–41 of HspB1 (and HspB6) (Fig 12A and 12B), contains a highly conserved sequence (that is found present in a subset of the vertebrate sHsp family as well as in close related homologous in *Drosophila melanogaster* [51]). This region is reported to possess a dual function and is able to inhibit the chaperone-like activity and at the same time participates in self-association of HspB6 (and probably other sHsp’s) [51].

The third region corresponding to residues 42–59 of HspB1 and HspB6 (Fig 12) seems to be important for stabilization of oligomeric structure of HspB1 [49] and for increased chaperone-like activity of HspB6 [51]. The C-terminal part of this region restricted by residues 42–56 of HspB1 (or residues 43–56 of HspB6, or residues 39–51 of HspB5) again participates in the interaction of HspB5 and HspB6 with target proteins [50, 51] and in regulation of oligomerization of small heat shock proteins tending to form large oligomers (like HspB1 and HspB5) [57].

Finally HspB1 contains a unique sequence, previously named P1 [49], that includes residues 59–77 (Fig 12). This region seems to play an important role in the formation of the large oligomers of this sHsp [58]. Although the sequence of this region is poorly conserved when comparing it to other vertebrate homologues, it appears rich in proline and in some species alanine residues.

To date the functional characteristics of the different parts of the N-terminal domain of the vertebrate sHsp have been determined either by truncation or deletion [51, 52, 57], by swapping regions between different sHsp [58] or by replacing of certain residues of the N-terminal domain by Cys followed by selective modification of the introduced Cys by electron paramagnetic resonance probes [49]. However, to our knowledge the effect of single point mutations on physico-chemical properties of HspB1 has not been systematically investigated.

The three congenital mutations characterized in this study all localize to the conserved region (Fig 12A and 12B). In particular Gly34 of human HspB1 is highly conserved being present in five other vertebrate sHsp (HspB2, HspB4, HspB5, HspB6 and HspB8) (Fig 12) [26]. Pro39 of human HspB1 is less well conserved across this subset of sHsp, but is also found in the equivalent position of human HspB2, HspB7 and HspB8 across the majority of species [26] (Fig 12A). Finally, residue E41 of human HspB1, like Gly34, is also highly conserved across homologues [26] (Fig 12).

Mutations in this conserved region are accompanied by similar changes in the structure and properties of HspB1. Indeed, all mutants form stable oligomers with the size slightly larger than the corresponding oligomers of the wild type HspB1 (Fig 5, Table 1). The increased size of the oligomers formed by the N-terminal mutants could be either due to an increased number of subunits involved in the formation of these assemblies or due to an increased size of the oligomer the core of which is packed less densely because of the unusually folded N-terminal domains of mutated HspB1. The last suggestion agrees with the data obtained by limited proteolysis. All mutants are less susceptible to proteolysis than the wild type HspB1 (Fig 4). Taking into account that the largest part of the sites most susceptible to chymotrypsinolysis are located in the N-terminal domain of HspB1 (Phe8, Phe19, Phe29, Phe33 and Trp45) we can hypothesize that mutations G34R, P39L and E41K somehow stabilize the structure of the flexible and therefore highly susceptible to proteolysis N-terminal domain of HspB1. It is postulated that the N-terminal domains are located in the center of sphere formed by large oligomers of αB-crystallin and participate in stabilization of this structure [12]. It is probable that mutations in the N-terminal domain of HspB1 enhance the interaction between N-terminal domains of neighbor subunits thus stabilizing the oligomeric structure of large oligomers of HspB1.

It is well known that the oligomeric structure of HspB1 is delicately regulated by phosphorylation of three sites (Ser15, Ser78, Ser82) located in the N-terminal domain and in the beginning of α-crystallin domain [43, 44, 52]. Incorporation of less than 1 mole of phosphate per mole of the wild type HspB1 is accompanied by partial or even complete dissociation of large oligomers and accumulation of small oligomers with apparent molecular weight ~90 kDa (Fig 7, S2 Fig). At the same time phosphorylation even up to more than 2.0 mole phosphate per mole of protein resulted in incomplete dissociation of large oligomers formed by G34R an E41K mutants and did not induce any significant changes in the oligomeric structure of P39L mutant (Fig 7). Thus, mutations in the N-terminal domain counteract the phosphorylation-induced dissociation of large oligomers which is assumed to be of great importance for effective functioning of HspB1 [49, 52]. This effect can be due to the increased interaction of the N-terminal domains between neighboring monomers, and hiding of these domains inside of the large HspB1 oligomers. In this respect it is worthwhile to mention that mutations P39C and E41C of the so-called 3D phosphomimicking mutant of HspB1 was accompanied by shifting equilibrium between small and large oligomers towards large oligomer of HspB1 [49]. Thus, the mutations G34R, P39L and E41K analyzed here partially or completely prevent dissociation of large oligomers induced by phosphorylation and to different extents increase the stability of these high molecular weight species.

Small heat shock proteins are able to form different heterooligomers with properties probably different from those of corresponding homooligomers [19, 59]. The wild type HspB1 formed two type of complexes with HspB6 with apparent molecular weights of ~100 and 300 kDa (Fig 8)[34, 45]. All N-terminal mutants of HspB1 were also able to form heterooligomeric complexes with HspB6, however in this case we detected only one type of complex, with an apparent molecular weight of ~400 kDa (Fig 8, Supplemental material S3 Fig). We also detected retardation of disulfide crosslinking of the so-called Cys mutant of HspB6 and the N-terminal mutants compared to those formed with the wild type HspB1 (Fig 9). Mutations in the N-terminal domain of HspB1 appear to affect the stability of the dimer interface in the heterodimers. The nature of this instability manifested by a change in subunit turnover or the increased possibility of forming another interface register at the dimer interface cannot be ascertained from the results presented.

By using three different model protein substrates we found that as a rule the wild type HspB1 possessed higher chaperone-like activity than any of the N-terminal mutants, however the difference in the chaperone-like activity was dependent on the nature of the model protein substrate (Fig 10). It is supposed that reversible dissociation/association of HspB1 oligomers plays an important role in its chaperone like activity [49]. Therefore the increased stability of the oligomers formed by the N-terminal mutants can modulate chaperone-like activity of HspB1. Moreover, the N-terminal domain contains several substrate-binding sites and therefore mutations of certain sites can differently affect chaperone-like activity with different model substrates, a phenomenon that was previously described [51]. It is worthwhile though to mention that the decrease of chaperone-like activity of the N-terminal mutants of HspB1 mutants is not due to a decrease of their hydrophobicity (Fig 11).

In summary, we conclude that the mutations of the N-terminal domain associated with hereditary motor neuron diseases likely stabilize the oligomeric assembly *in vitro* and therefore decrease or completely prevent phosphorylation-induced dissociation of large oligomers of HspB1 and modify its interaction with HspB6. In addition these mutations decrease the chaperone-like activity. It is possible that one or these effects, or a combination of thereof, alters the interaction of HSPB1 with proteins in the cell possibly leading to long term motor neuron diseases.

## Supporting Information

S1 Fig SDS-electrophoresis of the samples obtained after limited chymotrypsinolysis of the wild type HspB1 (A) and its P39L (B) and E41K (C) mutants. Time of proteolysis (min) is indicated above each track. The positions of the protein markers and their molecular weight (in kDa) are indicated by lines. Representative results of five independent experiments are presented.
(PDF)

S2 Fig Size-exclusion chromatography of the wild type HspB1 and its G34R, P39L and E41K mutants before phosphorylation (black curves) and after low levels of phosphorylation (red curves). The extent of phosphorylation is indicated on each panel.
(PDF)

S3 Fig MS spectra of tryptic peptides ALS^78^R (A), GPS^15^WDPFR (B) and QLS^82^SGVSEIR (C) before and after phosphorylation of the wild type (WT) or P39L (P39L) mutant of HspB1. (PDF)

S4 Fig Interaction of the wild type HspB1 and its G34R and P39L mutants with the wild type HspB6 analyzed by means of size-exclusion chromatography. On each panel the elution profiles of isolated wild type HspB1 (blue line), isolated mutants (green line) and isolated HpsB6 (dark yellow line) are presented. Elution profile of the mixture of two proteins preincubated at 4 and 42°C are shown as black and red lines respectively.
(PDF)

S5 Fig Disulfide crosslinking of the wild type HspB1 (A) and its G34R (B), P39L (C) and E41K (D) mutants. SDS-gel electrophoresis of the samples before oxidation and after different time of dialysis under non-reducing conditions. The time of dialysis (hours) is indicated above each track. The positions of the protein markers and their molecular weight (in kDa) are indicated by lines. Representative data of three independent experiments are presented.
(PDF)

S1 Table Molecular weights of tryptic peptides of the wild type HspB1 and its P39L mutant before and after phosphorylation. The values in brackets indicate calculated molecular weights of unphosphorylated and phosphorylated peptides.
(PDF)
