# Supplementary figures and images for: Characterization of Mutants of Human Small Heat Shock Protein HspB1 Carrying Replacements in the N-Terminal Domain and Associated with Hereditary Motor Neuron Diseases
Source: PLoS One. 2015 May 12;10(5):e0126248. doi: 10.1371/journal.pone.0126248 (PMC4429025; doi:10.1371/journal.pone.0126248)

A

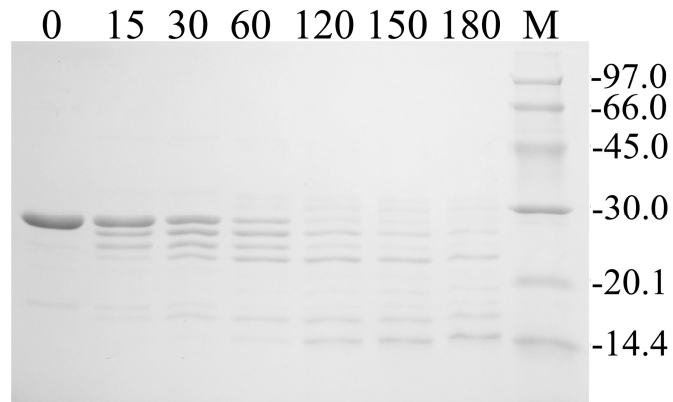

B

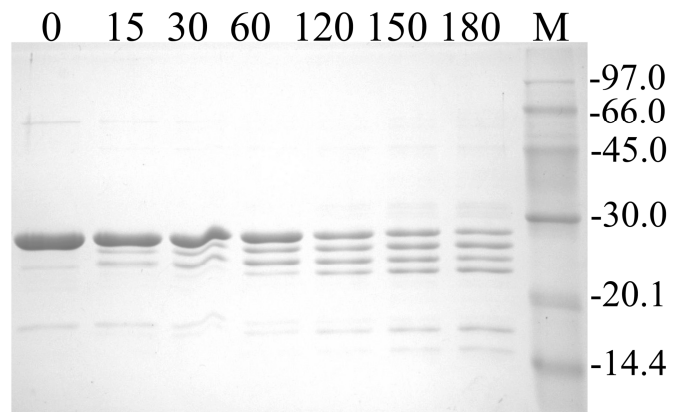

C

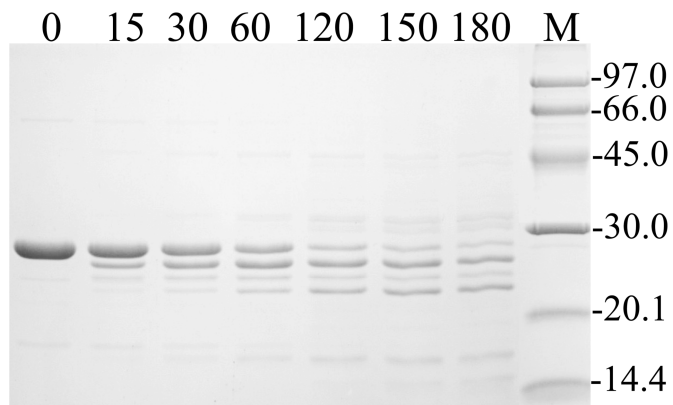

Supplement: S1 Fig — Time of proteolysis (min) is indicated above each track. The positions of the protein markers and their molecular weight (in kDa) are indicated by lines. Representative results of five independent experiments are presented. (PDF) [file pone.0126248.s001.pdf]

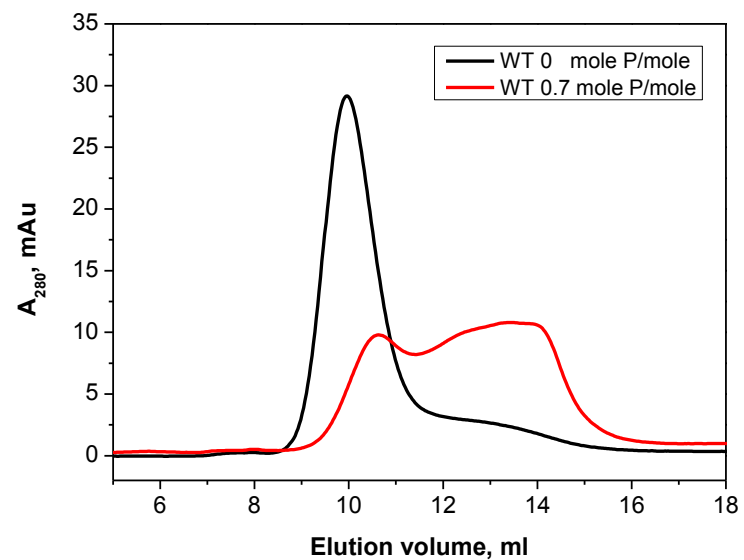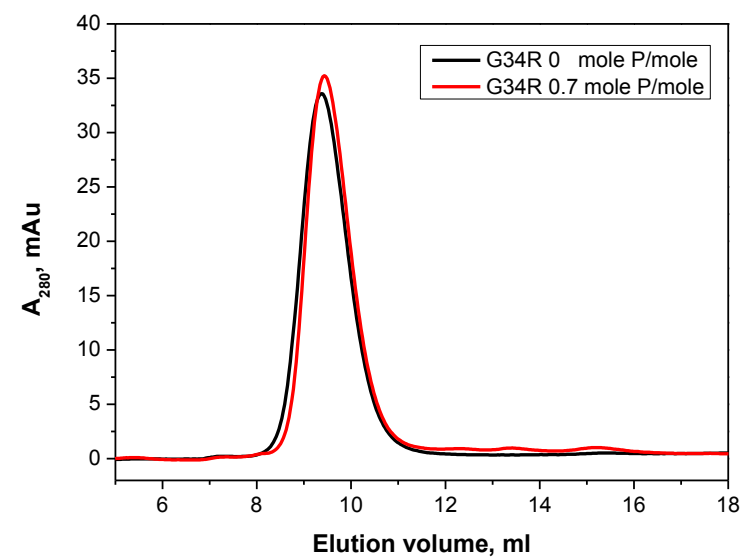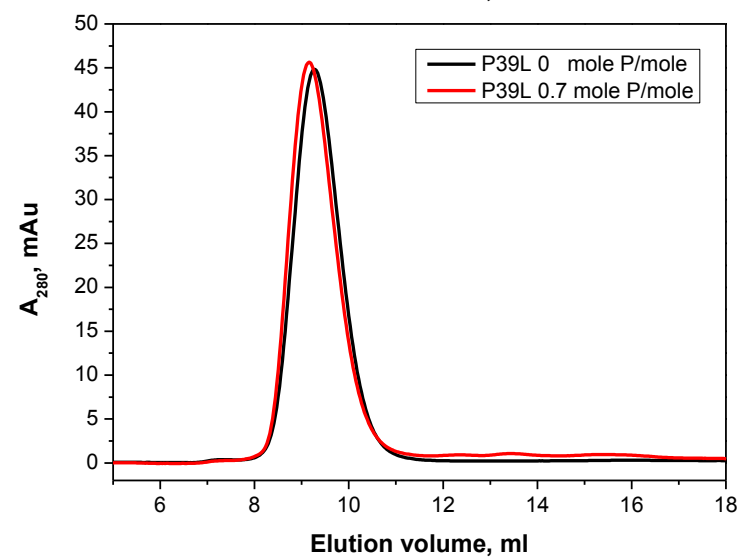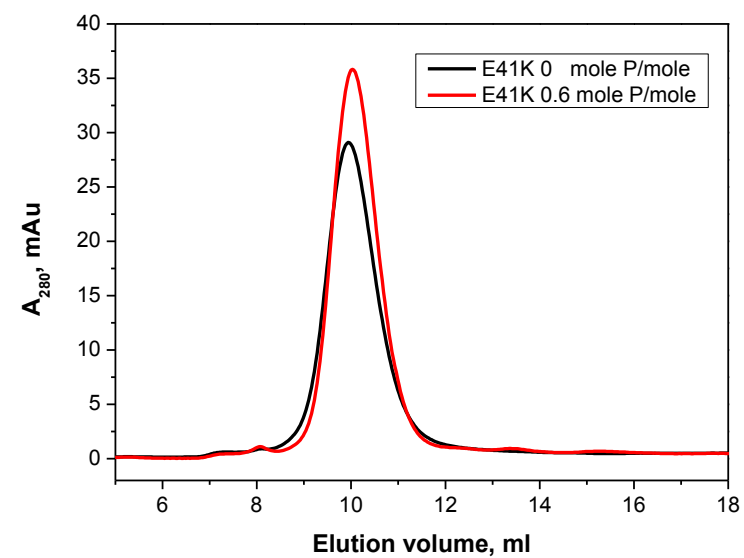

Supplement: S2 Fig — The extent of phosphorylation is indicated on each panel. (PDF) [file pone.0126248.s002.pdf]

A

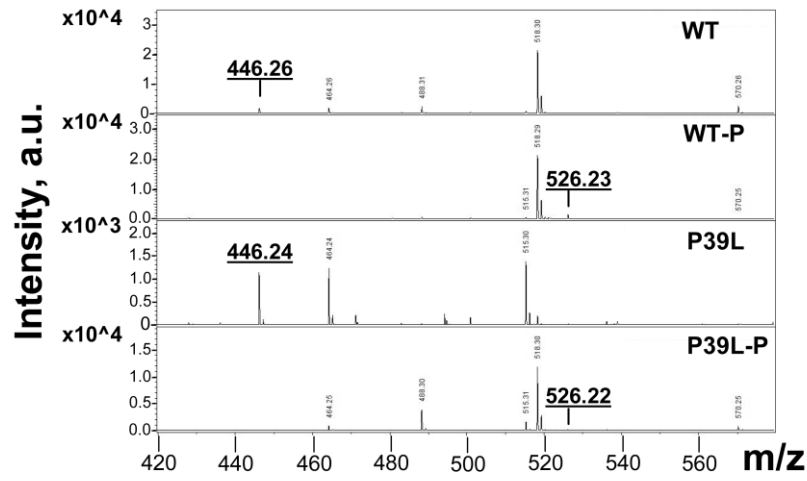

B

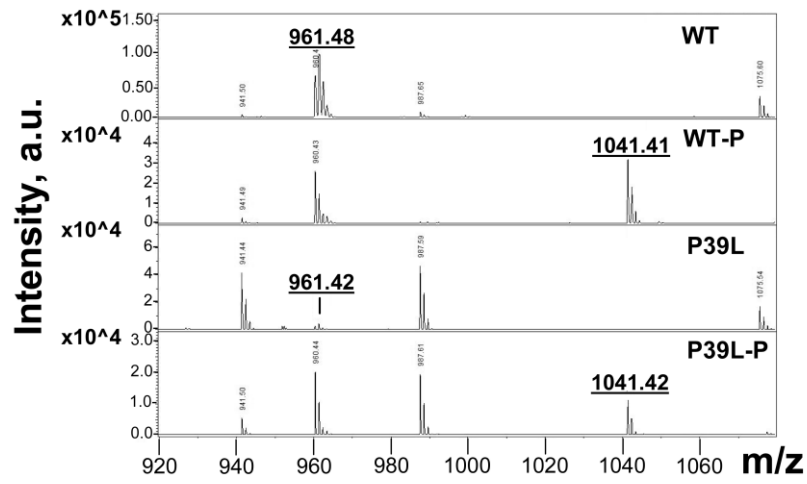

C

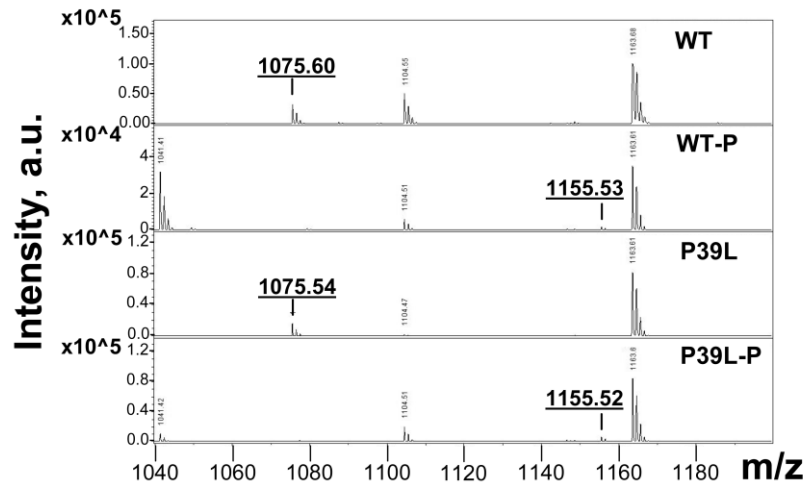

Supplement: S3 Fig — (PDF) [file pone.0126248.s003.pdf]

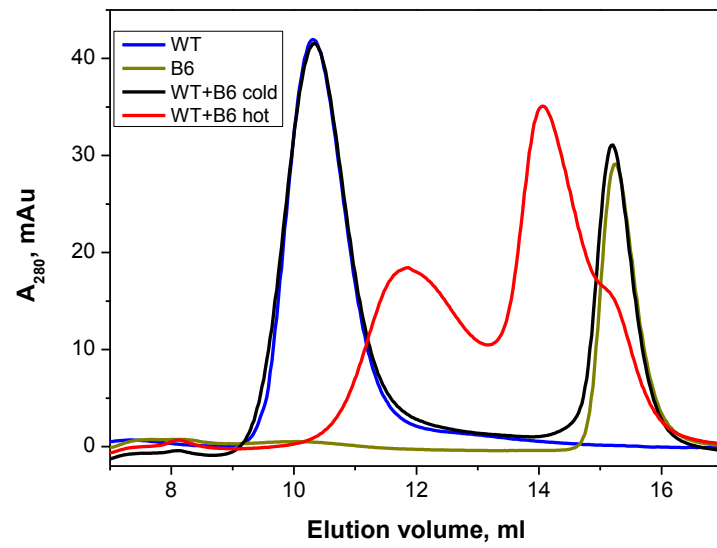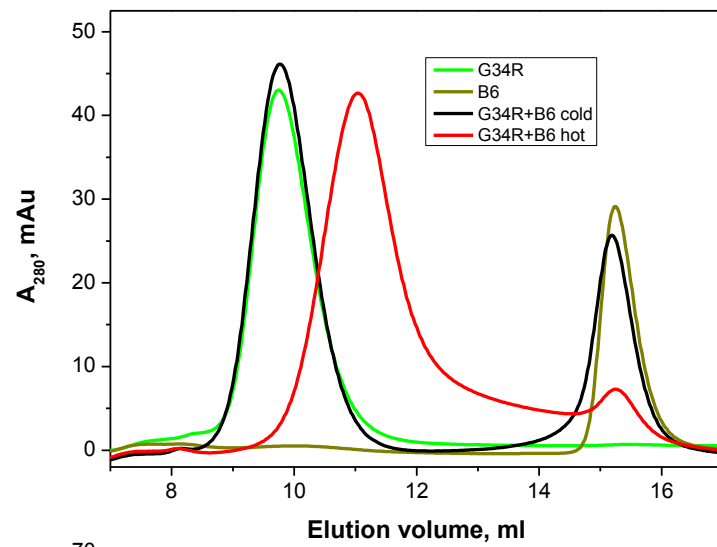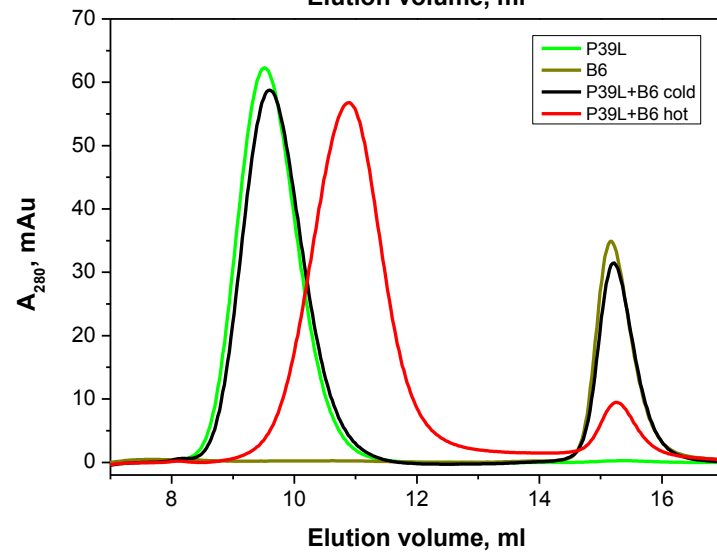

Supplement: S4 Fig — On each panel the elution profiles of isolated wild type HspB1 (blue line), isolated mutants (green line) and isolated HpsB6 (dark yellow line) are presented. Elution profile of the mixture of two proteins preincubated at 4 and 42°C are shown as black and red lines respectively. (PDF) [file pone.0126248.s004.pdf]

A

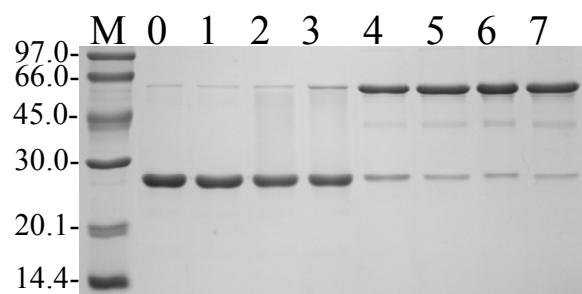

B

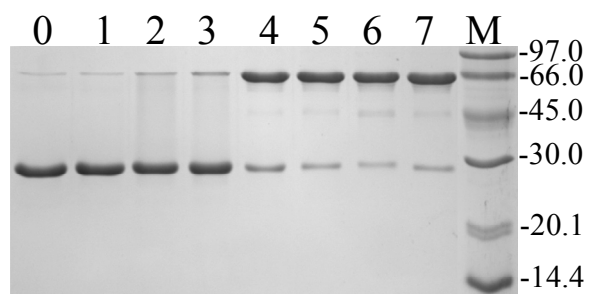

C

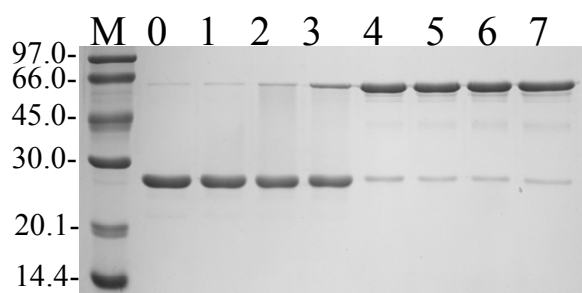

D

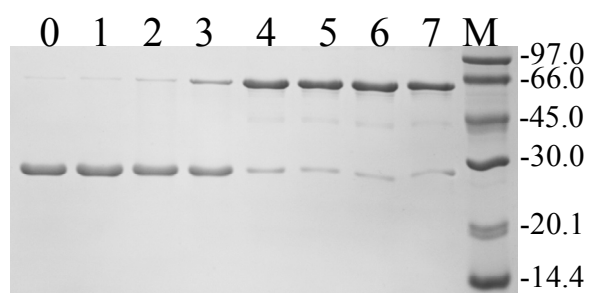

Supplement: S5 Fig — The time of dialysis (hours) is indicated above each track. The positions of the protein markers and their molecular weight (in kDa) are indicated by lines. Representative data of three independent experiments are presented. (PDF) [file pone.0126248.s005.pdf]
