# Supplementary material for: Characterization of Mutants of Human Small Heat Shock Protein HspB1 Carrying Replacements in the N-Terminal Domain and Associated with Hereditary Motor Neuron Diseases
Source: PLoS One. 2015 May 12;10(5):e0126248. doi: 10.1371/journal.pone.0126248 (PMC4429025; doi:10.1371/journal.pone.0126248)
Supplement: S1 Table — The values in brackets indicate calculated molecular weights of unphosphorylated and phosphorylated peptides. (PDF) [file pone.0126248.s006.pdf]

S1 Table. Molecular weights of tryptic peptides of the wild type HspB1 and its P39L mutant before and after phosphorylation. The values in brackets indicate calculated molecular weights of unphosphorylated and phosphorylated peptides.

Protein	GPS^{15}WDPFR		ALS^{78}R		QLS^{82}SGVSEIR	
**WT HspB1**	**961.48 (960.45)**	**None**	**446.26 (445.27)**	**None**	**1075.60 (1074.57)**	**None**
**WT HspB1 phosphorylated**	**None**	**1041.41 (1040.41)**	**None**	**526.24 (525.23)**	**None**	**1155.53 (1154.53)**
**HspB1 P39L**	**961.42 (960.45)**	**None**	**446.24 (445.27)**	**None**	**1075.54 (1074.57)**	**None**
**HspB1 P39L phosphorylated**	**None**	**1041.41 (1040.41)**	**None**	**526.23 (525.23)**	**None**	**1155.53 (1154.53)**
